# Population-based genetic risk prediction and stratification for ovarian cancer: views from women at high risk

**DOI:** 10.1007/s10689-014-9769-5

**Published:** 2014-11-13

**Authors:** Belinda Rahman, Susanne F. Meisel, Lindsay Fraser, Lucy Side, Sue Gessler, Jane Wardle, Anne Lanceley

**Affiliations:** 1 Department of Women’s Cancer, EGA Institute for Women’s Health, University College London, London, UK; 2 Department of Epidemiology and Public Health, Health Behaviour Research Centre, University College London, London, UK

**Keywords:** Ovarian cancer, Risk prediction, Risk stratification, Genetic testing, High risk, *BRCA1/2*

## Abstract

There is an opportunity to improve outcomes for ovarian cancer (OC) through advances in risk stratification, early detection and diagnosis. A population-based OC genetic risk prediction and stratification program is being developed. A previous focus group study with individuals from the general population showed support for the proposed program. This qualitative interview study explores the attitudes of women at high risk of OC. Eight women participated in one-on-one, in-depth, semi-structured interviews to explore: experiences of learning of OC risk, risk perceptions, OC knowledge and awareness, and opinions on risk stratification approach. There was evidence of strong support for the proposed program. Benefits were seen as providing reassurance to women at low risk, and reducing worry in women at high risk through appropriate clinical management. Stratification into ‘low’ and ‘high’ risk groups was well-received. Participants were more hesitant about stratification to the ‘intermediate’ risk group. The data suggest formats to effectively communicate OC risk estimates will require careful thought. Interactions with GPs were highlighted as a barrier to OC risk assessment and diagnosis. These results are encouraging for the possible introduction and uptake of a risk prediction and stratification program for OC in the general population.

## Introduction

 Ovarian cancer (OC) is the fifth most common cancer amongst women in the UK, and accounts for more deaths than all other gynaecological cancers combined despite a relatively rare age-standardised incidence of 17 cases per 100,000 females [1]. Early stage symptoms are few and non-specific, for example bloating and abdominal pain, and OC is often diagnosed at an advanced stage. Despite progress in surgical techniques and chemotherapeutic agents, 5-year survival rates remain low at around 40 % [2].

Current screening strategies for OC, including transvaginal ultrasound to visualise the ovaries and/or testing for biomarkers such as CA125, have not been successful in improving stage of diagnosis or mortality for OC. A recent meta-analysis of randomised controlled trials found that OC screening did not reduce mortality or the proportion of advanced stage diagnosis [3]. However the largest randomised controlled trial to date, the United Kingdom Collaborative Trial of Ovarian Cancer Screening (UKCTOCS) has yet to report. Differences between UKCTOCS and other OC screening trials may ultimately lead to the demonstration of a mortality benefit [4], but currently population-based OC screening is not recommended for asymptomatic women at average risk of the disease, although it may be part of the management plan for women at high risk [5]. Future screening approaches would benefit from identifying women who are at greatest risk of developing OC and targeting screening to this group [6, 7].

With the advent of next generation sequencing and the results of genome-wide association studies, knowledge of the genetic basis of disease susceptibility is rapidly expanding. There is potential to use this knowledge to classify individuals by their genetic risk for a particular disease [8]. This risk stratification approach could be applied to cancer screening. A population-based program for ovarian cancer risk prediction and stratification (PROMISE 2016 ‘Predicting Risk of Ovarian Malignancies, Improved Screening and Early detection) is under investigation. This program will develop and validate models for risk stratification, early detection and diagnosis of OC which incorporate clinical, epidemiological, proteomic and genetic data. These models will be used to predict each woman’s risk of OC, aiming to improve outcomes of the disease through prevention, screening and early detection. Although the program is still in the early stages of development and will be guided by our research, we present some details of the program below.

For model development, genetic data will involve genetic testing of high penetrance genes associated with hereditary breast and ovarian cancer, BRCA1 and BRCA2, as well as lower penetrance genes associated with ovarian cancer susceptibility, BRIP1, RAD51C and RAD51D [9–11]. Given the 10 % risk of ovarian cancer associated with Lynch syndrome [12], mismatch repair genes MLH1, MSH2, MSH6 and PMS2 will be included. Ongoing genotyping and genetic association studies are likely to yield other relevant genes and SNPs that could also be incorporated. Clinical and epidemiological data will include family history, environmental, hormonal and reproductive factors. For early detection and diagnosis, proteomic data will be used to improve current models of ovarian cancer screening and diagnosis.

This approach to OC risk stratification means women participating in the program will receive an estimation of OC risk and be categorised into one of three risk groups—low, intermediate or high. Thresholds for predicted risk for categorising individuals into a risk group will be based on retrospectively and prospectively validated models. Women will be offered risk-stratified information, support and risk management options. Some women at high risk may also be offered prophylactic measures such as bilateral salpingo-oophorectomy (BSO) to reduce their risk of OC. All women greater than 18 years of age will be eligible to participate; exclusion criteria includes previous oophorectomy, past history of tubal, ovarian cancer or primary peritoneal cancer, or personal history of genetic testing for ovarian cancer predisposing genes.

The feasibility of risk-stratified cancer screening is increasingly being explored, including the practicalities of implementation. The Collaborative Oncological Gene-environment Study (COGS) investigated the efficacy and cost-effectiveness of stratified screening for cancer using genomic information [13]. Through breast and prostate cancer modelling, personalised screening using age and polygenic risk detected the majority of cancers, while reducing the number of people screened. Although this report provides evidence for the potential benefits of risk-stratified screening, it also identifies the need for further research. Aside from the cost-effectiveness, utility, social and legal implications the COGS report identifies a ‘critical factor’ in the future introduction of risk-stratified screening, questioning whether ‘targeting resources according to risk is seen as compatible with the interests of the entire screening population’ (page 3). Given the novelty of a population-based risk prediction and stratification program for OC, there is a dearth of literature on this topic.

A Dutch study explored women’s attitudes towards genetic testing for breast cancer susceptibility with the aim of tailoring disease prevention strategies [14]. In general women had positive attitudes towards a breast cancer screening program based on genetic risk profile. This included genetic susceptibility testing prior to screening, as long as women who wanted to access screening were still able to do so despite being at low genetic risk. A number of issues were also raised around genetic testing, including personal autonomy, managing test results, potential discrimination and financial concerns relating to the cost of testing.

As part of the OC risk prediction and stratification program, we have begun to explore the opinions of the ‘entire screening population’. In an earlier focus group study we explored attitudes to PROMISE 2016 in women from the general population [15]. Like Henneman et al’s findings [14], participants expressed strong support overall for the proposed program, believing genetic testing for OC risk and subsequent stratified screening would be highly beneficial. However knowledge about OC and associated risk factors was consistently low. Risk perception for OC was also low and largely attributed to not having previously considered OC as a personal health threat. In contrast, there was awareness from all participants of the role genetics play in the development of cancer and the potential of genetic testing for OC risk was generally embraced. Knowledge about OC risk was seen as empowering, leading to possible preventive measures to ‘prepare for the future’. The concept of risk-stratified screening was also met with enthusiasm, although some concern was expressed that frequent OC screening for women identified at high risk may cause anxiety. For women identified at low risk, where screening would not be advised, a few participants expressed the desire for screening to be available on request, similar to the findings of Henneman et al.

Building on the results of our focus group study and the recommendations of the COGS report, this study explored the attitudes of women at high risk of OC to the idea of a population-based OC risk prediction and risk stratification program. With their greater knowledge and experience of OC, it was anticipated these women may offer insight into the acceptability and potential impact of taking part in the program. As the program is likely to identify a small number of women at high risk of developing OC, identifying the support and information needs to help women understand and adjust to their higher risk status is vital.

## Methods

### Sample

Ethical approval was obtained from the University College London Ethics Committee for non-NHS Research (project ID 3162/001). Women at high risk of OC, either due to a strong family history consistent with hereditary breast and ovarian cancer or BRCA1/2 mutation carrier status, were recruited to the study. As the aim of the program is early detection and prevention, only women without a personal history of cancer were invited. An invitation to participate in this qualitative study was placed on two UK ovarian cancer charity websites, The Eve Appeal and Ovacome (http://www.eveappeal.org.uk/; http://www.ovacome.org.uk/). Women interested in being interviewed were invited to contact the research team directly to find out more about the study.

### Procedure

Women who contacted the research team were sent a detailed information sheet. Interviews were conducted in person or by telephone depending on the woman’s preference. Ten women participated in one-on-one, in-depth, semi-structured interviews. An interview guide was used, leaving wording and sequence of topics open and with probes to elicit more information if needed. The discussion topics included: experiences of learning of OC ‘high risk’, risk perceptions, OC knowledge and awareness, opinions on risk stratification and opinions on risk management options. An explanation of the risk stratification approach and risk management options was given to participants (Table 1). Interviews were digitally recorded and transcribed verbatim. Participants also completed a consent form, brief demographic questionnaire and cancer family history form. Data for two of the 10 women interviewed were excluded because it was established during the interview that one had a diagnosis of breast cancer, and one had previously had predictive BRCA1/2 testing and received a negative result.

**Table 1:** Information provided to participants on risk stratification and risk management options

*Opinions on a risk prediction program using risk stratification approach*
Risk stratification means that women can be grouped based on their likelihood of getting ovarian cancer. Women can be described as having a low, intermediate or high risk. The level of risk is based on a woman’s genetic risk and other risk factors. Identifying genetic risk involves having a blood test. Identifying other risk factors would involve filling in questionnaires about cancer family history, background and health information. Scientists can then put all of this information together and estimate whether a women is at low, intermediate or high risk. It is estimated that 50–60 % of women will be at low risk, 30–45 % at intermediate risk, and 4–7 % at high risk

*Opinions on possible risk management options*
Depending on a woman’s risk level (low, intermediate or high), she would receive different levels of risk management for ovarian cancer. Women at low risk would receive information telling them that they are at low risk and that they do not need further monitoring. This information would also let low-risk individuals know about symptoms of ovarian cancer. Women at intermediate risk would receive screening every year (screening involves a blood test to check for levels of the biomarker CA-125 followed by transvaginal ultrasound). Those at high risk would be screened every 4 months. High-risk women may also be referred to a specialist to discuss risk-reducing surgery

### Data analysis

Thematic analysis was used to analyse the interview transcripts. This method identifies, analyses and reports patterns or themes within the data [16]. The process of analysis involved several stages, beginning by reviewing the data and noting initial ideas. Once familiarised with the data, initial codes were then generated in a systematic manner for the entire data set. Codes were collated into potential themes, where similar but separate codes were combined and refined. Themes were reviewed, refined and organised into a final theme list. Transcripts were coded by BR using the qualitative data analysis software QSR NVivo 10. To increase rigour all transcripts were also coded by another member of the research team (SM); any differences were discussed until agreement was reached.

## Results

Participants were aged between 25 and 58 years, and had a variety of experiences in terms of genetic testing, risk-reducing surgery and OC screening (Table 2). All had a family history of at least one first degree relative with OC or multiple first and second degree relatives with breast cancer and/or OC.

**Table 2:** Participant characteristics (n = 8)

Participant	Age	Genetic testing	Genetic testing result	Risk-reducing surgery		Screening
				BSO	Mx	
HROC_02	38	No	–	Yes	No	No
HROC_03	42	Yes	No mutation	Yes	No	No
HROC_04	45	Yes	No mutation	Yes	No	Yes—until BSO
HROC_05	35	Yes	BRCA2 +ve	No	Yes	Yes
HROC_06	37	Yes	BRCA1 +ve	No	No	No
HROC_07	58	No	–	No	No	Yes
HROC_08	29	Yes	BRCA1 +ve	No	No	No
HROC_10	25	Yes	BRCA1 +ve	No	Yes	No

BSO = bilateral salpingo-oophorectomy, Mx = mastectomy, Screening = transvaginal ultrasound and/or CA125 biomarker, +ve = mutation positive

### Ovarian cancer perceptions

OC was often described by participants as an ‘unknown’ disease. Prior to the diagnoses in their family, participants had little or no knowledge about OC or possible risk factors. For the participants who were BRCA1/2 mutation carriers with a family history of breast cancer, the risk of OC was not known until it was disclosed during the genetic testing and counselling process. Learning about OC risks was unexpected, with these participants describing the experience as a ‘shock’ or ‘surprise’.

So yeah, hearing that I did have that risk, like I said, that was quite a shock to me. I hadn’t really linked the two, I guess because we didn’t know for a while that it was a genetic thing. I always thought it was breast cancer that came my way. I didn’t realise that I was such high risk for ovarian. (HROC_05)

Coupled with the lack of information available in the general community, participants were also fearful of OC. This was exacerbated by the lack of effective methods to detect the cancer; giving the impression that OC is an ‘invisible’ disease which may not be identified until an advanced stage due to the vague symptoms associated with OC such as bloating and pelvic or abdominal pain. When compared to breast cancer, participants felt OC was a more complex and worrying disease. Despite BRCA1/2 carriers being at higher relative risk for breast cancer (up to 80 %) compared to OC (with risks of up to 40 %) [17], participants felt less concerned about breast cancer because they perceived there was greater awareness about the disease, and early detection and screening methods were more effective.

I think also, as well, there is so much more information out there about breast cancer that it doesn’t frighten me as much, I think because it is more easily detectable. I think the whole thing about the potential for an ovarian tumour, the whole thing that frightened me so much about it was that it is so hard to detect. You know, it is not something that would show up on a smear, you wouldn’t necessarily have lumps protruding from your stomach, it is very, very difficult to detect it and I think that was a big part of the fear factor for me, was I could have this, I could already have this and it would be relatively well advanced and I wouldn’t necessarily know, you know. Whereas in the case of breasts, you know, if you are doing the checks, you are going to find a lump. (HROC_02)

### Attitudes towards PROMISE 2016

There was a strong positive response to the idea of a population-based risk prediction and stratification program. Participants felt the main benefit of the program would be to provide accurate information and support regarding OC risks, either by reassuring women who mistakenly believed they were at increased risk or by providing effective clinical interventions (surgery/screening) for those who were in fact at increased risk. The program would also play a key role in raising women’s awareness about the disease.

I think if you are looking around your family and thinking there’s so many cancers, you know, what have I got, what am I going to get, I think it might also be a reassurance that no, these are just really bad luck and it’s not necessarily something that’s destined to come your way. It could be quite a good reassurance for people whose risks they perceive to be higher than they actually are. (HROC_05)

There were varying opinions on how the program would be received by the female population. A number of participants felt that the majority of eligible women would be interested and willing to be involved in the program to learn their risk for OC, but others felt that interest and motivation would be low given the lack of awareness about OC and associated risk factors. Participants felt that a family history of cancer or existing worries about developing OC would motivate participation.

The idea of women being stratified into low and high risk OC groups was well received. Participants felt that women in the low risk group would experience a sense of relief, particularly if they had anticipated being at higher risk for OC. For women identified as being in the high risk group, knowing this information and having regular screening or testing was seen as reassuring, with surveillance for early detection acting as a safety net. The concept of an intermediate risk group was met with some hesitancy by the participants. A number of women were unsure of what benefit having this information would provide. The perception was that individuals in this risk category may be concerned at being at increased risk compared to the low risk group, but without being offered the surveillance or prevention options available to those in the high risk group.

That’s the thing. If you are high risk, also, then you can then go down my route. If you are low risk you are just becoming more aware of just general health issues. But if you are intermediate I don’t know what the benefit would be to know really because you wouldn’t necessary get the… well, I don’t know, maybe you would. Would you go and have a hysterectomy? I don’t know. That would be my question. (HROC_06)

In terms of the suggested screening and interventions for the risk-stratified groups, participants felt that for the low risk group receiving information about OC, in particular potential symptoms, was sufficient. Interest in annual and 4-monthly screening, respectively, for the intermediate and high risk groups was tempered by concerns about the efficacy of screening.

### Knowledge and information

Knowledge was seen as learning about genetic risks for OC through genetic testing or assessment of family history, culminating in receiving a risk estimate of developing the disease. Participants were unanimous in their desire to have this information, describing knowledge as power. They also felt strongly that this knowledge should be used to take action and reduce their risk of developing cancer; an opportunity that other family members had not had.

Gosh, I mean, I had the option before I went for the genetic test, obviously, not to go for it, but, for me, no, I wanted to know. It was forewarned is forearmed. I can do something about it. (HROC_05)

Given this desire for knowledge, participants insisted that the risk prediction program must provide information not only regarding the risk-management aspect of the program, but about OC itself. In their previous experiences they had struggled to find accurate and reliable information regarding risk factors, symptoms, screening and preventive options. As a result participants described wanting as much information as possible. They felt this information would need to be an integral part of the risk prediction program to allow women in the population to make an informed decision about participation in the program.

If you know what you are looking out for, you know, it could be that you do actually just have IBS, but equally, you might not and I think the more women know and the more information they have, they can make the rational decisions themselves about do I need to seek medical help here or, you know, can I maybe leave it a little while and see if simple analgesia and a hot water bottle makes me feel better? But I think the more information people have, by far and away the better. (HROC_02)

### Risk communication

The participants who had previously had genetic testing and genetic counselling and been found to carry a BRCA1/2 mutation recalled receiving risk estimates for breast cancer and OC described as a percentage risk. In general this format was well received, being described as easily understood and interpreted. They were also provided with age-related risks, a specific age or age range at which OC risk becomes significant. Age-related risk information was perceived as very helpful—participants described being able to compartmentalise their risk until they reached their ‘at-risk age’, deferring their cancer worries to a specific time in the future. Those who had less formal risk assessment recalled their risk described as ‘10 times higher than the general population’, or a ‘1 in 8’ chance of developing OC.

I am not saying it’s right or wrong, but I have always been told, “You don’t need it. You just need to know your at-risk age. You have got the gene. You are high risk, we know that. We need to work out your at-risk age category.” So, for me, it’s, like, it’s very early 40s and that’s what I have always worked on. (HROC_06)

Participants had many suggestions about how risk estimates should be communicated in the OC risk prediction and stratification program such as numeric risk estimates, preferably percentages, and descriptive estimates. Comparisons to general population risks were recommended to help women put their risk in perspective. The challenge of using only one risk format for a large diverse female population was acknowledged and reflected in the lack of consensus about what the preferred format should be. There was agreement that the ‘higher’ risk estimates such as 1 in 10 would be more meaningful than trying to interpret ‘lower’ risks such as 1 in 1,000 or 1 %. Clear distinctions in risk estimates between the three risk groups—high, intermediate and low—would be important so that women would be aware of not only their risk estimate but also risk ‘classification’.

Because if you are 1 in a 1,000 and you are low risk, it does sound, oh, it’s never going to happen to me, it’s never going to happen to the 1 in a 1,000, it’s never going to happen to me. Whereas if you are intermediate risk then it needs to be a bit more clear that, you know, it is a higher risk. (HROC_06)

### Psychological support

The interviews showed that participants employed individual strategies to cope with their high risk status. For some, there was a sense of wanting to do as much as possible to reduce their cancer risk, either through surgery or screening. Participants who had undergone risk-reducing surgery (either mastectomy or oophorectomy) felt reassured that their cancer risks had been reduced by their actions, despite any complications from surgery and the challenges of managing menopausal symptoms.

I just have to turn it into a positive thing and, you know, even the normal, you know, people without the gene have got a higher risk than me now. So it’s a good thing and something that my mum and the rest of the family couldn’t have done. (HROC_10)

Other participants who had not undergone BSO described the importance of not dwelling on OC risks; while they acknowledged that risks remain, rumination was not seen as a helpful strategy.

The things that I can do, I have done, but I am not prepared at the moment to think about the option of surgery again until it becomes a greater risk… I feel like I have done what I can at the moment but I don’t want to, kind of, tie myself up in mental knots by having to think about it too much now. I am doing all I can realistically, I think. (HROC_05)

It was encouraging that the participants in this study were able to use different coping strategies to manage their high risk status. Although the risks remain ‘at the back of the mind’, they were able to continue their lives. Given the psychological burden most women at high risk for OC may face, it is not surprising that all participants spoke about the need for psychological and emotional support to be provided within the program. Ideally support would be available before and after receiving OC risk estimates, with a preference for face-to-face interactions with either a psychologist or genetic counsellor.

It is, and I think based on my own experiences and going by what I could have done with and didn’t have access to, I think for women at all levels of risk, the one thing I would say would be ensure that there is some form of emotional support, even if, as part of the study, you made it mandatory. (HROC_02)

### Interactions with health professionals

Interactions with health professionals (HPs) were an integral part of all participants’ narratives of their OC experiences. Interactions with General Practitioners (GPs) were often described as particularly distressing, with missed or delayed diagnoses attributed to the GPs lack of interest in, or knowledge of, OC. GPs were often perceived as barriers to further investigations or referral to specialists. Some women described the ‘battle’ they faced for requests to see a gynaecologist or for CA-125 testing. There was also sense of paternalism embedded within the interactions—women were told ‘not to worry’ and their symptoms were often dismissed as overreactions. There is clearly a need for better training and education for health professionals, in particular GPs, about OC.

But it does come down to a doctor in a lot of ways but I think it’s the GPs often because that the first port of call before you even get to there. I mean, my mother must have gone to a GP many times before she got to see a consultant, many times, many, many, many times. So it’s the GPs, really, who can be a stumbling block and it’s often about… What I feel personally, is there’s a lot of emphasis on things like heart disease and diabetes and obesity and the, kind of, women issues tend to get just […dismissed]. (HROC_04)

## Discussion

The prospect of genetic risk stratification to inform screening programs is becoming increasingly viable given the advances in genetic technology. Like our earlier focus group study with women from the general population [15], women at high risk for OC had positive attitudes towards the idea of a population-based risk prediction and stratification program. It is encouraging that women with different experiences and knowledge of OC are supportive of the program.

Although the proposed program was well received, there was a lack of consensus amongst participants in how they felt it would be received by the general population. Some felt the main motivating factor for uptake would be pre-existing worry for cancer, perhaps reflecting their own perspectives of being at high risk for OC. The influence of cancer worry has previously been described in the cancer screening and genetic testing literature. Women who accept predictive BRCA1/2 genetic testing tend to have significantly higher levels of cancer worry than women who decline testing [17]. Cancer worry also plays a role in cancer screening behaviours; a review by Hay concluded that participation in cancer screening is facilitated by cancer worry both for individuals from the general population as well as those at high risk of cancer [18]. This has important implications for the potential implementation of the program. Existing national cancer screening programs such as mammography for breast cancer show that uptake by eligible women in the UK is relatively high at 73 % [16]. Although stratified screening would be expected to reduce the overall number of people who have screening by targeting those at greatest risk, it may in fact increase uptake by those who are eligible. The question remains whether providing genetic information about risk of cancer development will increase participation and adherence to a screening program.

The majority of participants felt the main benefit of the program would be to (1) provide reassurance for women classified into the low risk group and (2) reduce worry for women at high risk who could be appropriately managed with risk-reducing surgery or regular screening. Implicit in this belief is that women who participate in the program will feel, to some degree, at risk for OC. However a survey of OC risk perceptions among women participating in the UKCTOCS trial found that around 40 % of women accurately estimated their lifetime risk of developing OC, around 50 % underestimated their risk, while less than 10 % overestimated the risk [18]. Similar estimates of risk were found in a large-scale survey of Australian women [19]. In general it seems women from the general population have accurate or lower perceptions of risk regarding OC development in their lifetime. There is a tendency for individuals to believe they are less at risk compared to their peers to a range of risks, including health risks. Termed unrealistic optimism by Weinstein, it may explain some of the optimistic bias seen in OC risk estimations [20]. In contrast, previous studies have shown that a significant proportion of women at *increased* OC risk due to family history or BRCA1/2 mutation overestimate their risk [21, 22]. The reassurance described by participants may be most effective for the small group of women overestimating their risk of OC and those identified at high risk.

Participants also felt a significant benefit of the program would be to raise awareness about OC. A number of studies, including the earlier focus group work, have identified low awareness of OC symptoms and risk factors in women in the general population [15, 19, 23]. Participants in the current study described their own lack of knowledge regarding OC symptoms, and their struggle to find accurate and appropriate information. They felt that a large scale effort is needed to educate the public about OC, so it becomes integrated into the ‘female psyche’ as has been the case for breast cancer.

A significant part of PROMISE 2016 will involve the communication of OC risk estimates to the public. In this study we referred to three risk categories—low, intermediate and high—that may be used for risk stratification. It has been suggested that the risk information communicated was simplified by participants with only the ‘gist’ of the information being recalled, i.e. ‘low risk’ versus ‘high risk’ and therefore the ‘intermediate risk’ is ignored [24]. A binary understanding of risk, e.g. something will or will not happen, or low vs high risk, has also been described in the genetic counselling literature as a response to receiving genetic risk information [25]. This was reflected in the current study, as one high risk participant said, ‘*Because even me, I think I am 65* *% risk and although that is high, in a way it almost doesn’t sound high because it almost sounds like, oh well, it’s almost 50/50′*. Another participant commented, *‘You see, and the other thing about risk is, in the end it doesn’t matter what the percentage is or what your chance is, what the risk is… You know, you can’t get 10* *% of ovarian cancer. You either get it or you don’t.’* Given the responses of women at high risk to the proposed intermediate risk category, coupled with the lack of interest in the lower risk categories by participants in the focus group, the planned approach to risk categorisation may need to be modified. Using a binary approach for risk stratification may be more suitable. For example two categories of low*er* and high*er* risks to differentiate between women with risk estimates low enough not to warrant further worry about OC (outcome: reassurance) and those with estimates high enough to be under surveillance (outcome: screening).

Methods for communicating the individual estimates of OC risk also need to be considered. Despite the preference for receiving risk information as a percentage risk, studies have shown that people struggle to understand numerical probability statistics, with comprehension and interpretation influenced by their level of numeracy [26]. However verbal expressions to convey probabilities can be interpreted too subjectively [27]. The importance of effective risk communication cannot be underestimated; studies have shown that risk perception is a better predictor of behaviour rather than the actual risk communicated [28]. There is evidence that risk perceptions can influence screening behaviours and medical decisions [29, 30]. In the present study, some participants indicated a preference for estimates presented as percentages or proportions, while others felt concerned that providing a numeric risk would be frightening and preferred descriptions or comparisons to the general population. These results highlight individual preferences for receiving genetic risk information. Developing formats of communicating risk estimates that are well received and comprehensible to a large and varied population is a key issue for the successful implementation of the program.

Fear of OC, attributed to its ‘unknown’ and invisible nature was often described by participants. They felt non-specific OC symptoms made it difficult to identify which are ‘real’ symptoms that should be investigated further, leading to diagnostic delay [31–33]. Delays were a prominent theme in the interviews, with numerous descriptions of the difficulties participants faced in their interactions with health professionals, in particular the GPs responsible for their own or their relative’s care. These experiences were distressing and it seems that these challenging interactions are not uncommon in women at high risk for OC [34]. Delays experienced by British women in obtaining an OC diagnosis are also compounded by doctor or health service delays [32]. Raising awareness about OC is not only important in the general population, but also within the medical profession. Future research regarding this program needs to consider approaches to improving interactions between providers and patients, as well as education programs targeted at GPs.

Despite clinical advances in understanding the pathogenesis of OC and development of targeted treatments, outcomes remain poor. Current OC screening involving CA125 biomarkers and transvaginal ultrasound have not yet shown sufficient sensitivity or specificity to be used clinically. Research now turns to novel methods of early detection and prevention. By combining increasing knowledge on underlying genetic susceptibility of OC, biomarker discovery and validation, modelling and health behaviour research, PROMISE 2016 endeavours to translate these advances into a population-based intervention. Although the program is at an early stage of development, risk stratification for OC has the potential to reduce the number of OC diagnoses by identifying women at high risk and offering risk-reducing management options. When high risk of OC is due to carrying a highly penetrant gene mutation, identification of this risk benefits not only the woman who participates, but may also have a cascade effect for at-risk relatives. From a screening perspective, until data from UKCTOCS are reported it is difficult to comment on the benefit of identifying women at increased risk, however advances in proteomics are being used to develop accurate biomarkers capable of significantly improving early detection and diagnosis. The response of participants to the proposed intermediate risk group in this study was invaluable for our understanding of perceived utility of risk categorisation, and will influence the development and implementation of the program in the future.

## Limitations

We acknowledge that this is a small study involving eight participants and is by no means an exhaustive exploration of the topic. There was a variety of opinions and experiences across the participants, but there was also consensus in support for and attitudes towards the risk prediction and stratification program. This level of thematic representation across the data set is entirely acceptable according to Braun and Clark [16]. Recruiting women to participate from the ‘public’ setting through OC charity websites, and not from a hereditary cancer clinic, proved more challenging than anticipated. It may have been that the majority of women who access the websites have already been affected by cancer, which was the only exclusion criterion of this study.

## Conclusion

What is clear from these eight interviews is that the process of learning about OC risk is a long and often fraught journey. Many women had witnessed their close relatives suffer and, in most cases, eventually succumb to the disease. Barriers between them and their health professionals meant participants felt they struggled for their OC concerns to be acknowledged and addressed. Despite variation in age, family history and OC experiences of the participants, there was strong consensus amongst the group that a population-based risk prediction, stratification and screening program for OC should be introduced. The main anticipated benefit was the relief and reassurance it would provide to the majority of the population who would be classified at low risk for developing OC. The call for more information regarding OC as well as the provision of psychological and emotional support, and the need for this to be provided by PROMISE 2016 was highly informative. The lack of consensus for how to communicate OC risk estimates to the public clearly indicated the need for more research in this area.

Although these results are encouraging, the task now turns to exploring the logistics of program implementation where uptake rates for genetic testing are known to be significantly lower than reported interest and intention levels [35–37]. This preliminary stage of research involves exploratory work with stakeholders, in this case the ‘entire screening population’, to learn their views and attitudes towards the proposed program. To further our understanding of attitudes and acceptability, interviews with another key group of stakeholders, health professionals, will also be undertaken.
